# Transoral Laser or Robotic Surgery Outcomes for Oropharyngeal Carcinoma: Secondary Analysis of the PATHOS Randomized Clinical Trial

**DOI:** 10.1001/jamaoto.2024.3371

**Published:** 2024-10-10

**Authors:** James T. O’Hara, Christopher N. Hurt, Kate Ingarfield, Joanne M. Patterson, Katherine Hutcheson, Joanna E. Canham, Lisette S. Nixon, Christie D. Heiberg, Sean Johson, Mererid Evans, Terry M. Jones

**Affiliations:** 1 Population Health Sciences Institute, Newcastle University, Newcastle, United Kingdom; 2 Department of Otolaryngology–Head and Neck Surgery, Newcastle-upon-Tyne Hospitals NHS (National Health Service) Foundation Trust, Newcastle, United Kingdom; 3 Southampton Clinical Trials Unit, Southampton General Hospital, Southampton, United Kingdom; 4 Centre for Trials Research, Cardiff University, Cardiff, United Kingdom; 5 Liverpool Head and Neck Centre, University of Liverpool, Liverpool, United Kingdom; 6 Department of Head and Neck Surgery, University of Texas MD Anderson Cancer Center, Houston; 7 Division of Radiation Oncology, University of Texas MD Anderson Cancer Center, Houston; 8 Division of Cancer and Genetics, School of Medicine, Cardiff University and Velindre University NHS Trust, Cardiff, United Kingdom

## Abstract

**Background:**

Transoral robotic surgery (TORS) or transoral laser microsurgery (TLM) offer excellent oncological outcomes for oropharyngeal squamous cell carcinoma caused by human papillomavirus (HPV) infection. TORS may offer greater margin clearance around tumors than TLM.

**Objective:**

To determine whether the differing energy sources used and surgical technique of TORS or TLM is associated with postoperative early swallowing function, feeding tube use, and specific factors related to quality of life.

**Design, Setting, and Participants:**

This prespecified cohort study was performed within the Postoperative Adjuvant Treatment for HPV-Positive Tumours (PATHOS) randomized clinical trial at 40 centers in the UK, Germany, France, the US, and Australia between November 1, 2015, and August 31, 2023. PATHOS trial participants with HPV-positive oropharyngeal carcinoma of stages T1 to T3 and N0 to N2b M0 (TNM7) who underwent TLM or TORS were eligible. Of 989 consecutively recruited patients on the PATHOS trial, 508 were eligible for this substudy.

**Exposures:**

The exposure of interest was TORS or TLM.

**Main Outcomes and Measures:**

Preplanned outcome measures included nasogastric tube insertion rates within 4 weeks after surgery, length of in-hospital stay following surgery, specific scales from the MD Anderson Dysphagia Inventory (MDADI), 35-item European Organization for Research and Treatment of Cancer Head and Neck Questionnaire (H&N35), and 30-item Quality of Life Questionnaire (QLQ C30), water swallow test results, and videofluoroscopy scores.

**Results:**

Of the 508 patients included in the analysis (390 [76.8%] male; median age, 58.3 [IQR, 52.8-63.6] years), 195 had TLM and 313 had TORS. Nasogastric tube insertion rates were higher after TORS than TLM (85 of 189 [45.0%] vs 10 of 126 [7.9%]; adjusted odds ratio [OR], 4.41 [95% CI, 1.01-19.32]). Mean scores favored TLM with small effect sizes in all MDADI domains and the H&N35 swallowing item at 4 weeks after surgery; between-group difference for the MDADI composite score was −4.89 (95% CI, −8.27 to −1.50); for the MDADI physical functioning score, −6.37 (95% CI, −10.15 to −2.59); for the MDADI global score, −10.02 (95% CI, −16.50 to −3.54); and for H&N35 swallowing score, 7.24 (95% CI, 2.17-12.30). No other measures showed evidence of clinically meaningful differences.

**Conclusions and Relevance:**

In this cohort study, functional outcomes were moderately less impaired 4 weeks following TLM compared with TORS. Once the longer-term outcomes for these patients are known, these findings could aid the design and use of future head and neck–specific surgical robots.

**Trial Registration:**

ClinicalTrials.gov Identifier: NCT02215265

## Key Points

**Question:**

What are the differences in early postoperative functional outcomes between transoral laser microsurgery (TLM) and robotic surgery (TORS) for human papillomavirus–positive oropharyngeal carcinoma?

**Findings:**

In this cohort substudy that included 508 patients within a randomized clinical trial, nasogastric tube insertion rates within 4 weeks after surgery were significantly higher after TORS than TLM (85 of 189 [45%] vs 10 of 126 [8%]). Mean scores on patient-reported outcome measures indicated significantly less impairment at 4 weeks following TLM.

**Meaning:**

These findings could influence the design and use of future head and neck–specific surgical robots.

## Teaser

This cohort study explores whether the different energy sources used and differing surgical philosophy of transoral robotic surgery and laser microsurgery are associated with postoperative early swallowing function, feeding tube use, and specific factors related to quality of life among patients with human papillovirus–positive oropharyngeal squamous cell carcinoma.

## Introduction

Oropharyngeal squamous cell carcinoma caused by human papillomavirus infection (HPV-positive OPSCC) is increasing in incidence in the UK and other high-income countries.^1^ It tends to affect younger patients and has a better prognosis than other head and neck cancers. Treatment options include either radiotherapy with or without chemotherapy or surgery. Small- and intermediate-volume primary tumors (stages T1-T2 and some T3) can be resected using transoral surgery. Both transoral laser microsurgery (TLM) and transoral robotic surgery (TORS) offer excellent oncological outcomes. Disease-specific survival rates in excess of 93% for HPV-positive OPSCC have been reported by Dalton et al^2^ and O’Hara et al^3^ in the UK. TLM became widely used in the 1990s and 2000s and uses a carbon dioxide laser to undertake a transtumoral resection to remove a cancer in 2 or more planned pieces,^4^ with the aim of preserving as much adjacent normal tissue as possible without compromising oncological safety. Specifically, in performing an oropharyngectomy, the superior constrictor is not removed in total or in part unless tumor invasion dictates. The extent of resection is therefore determined by the size and anatomical orientation of the tumor.

TORS has become more widely used than TLM to treat HPV-positive OPSCC, with a rise in the number of institutions having access to surgical robots.^5^ In contrast to TLM, TORS uses monopolar diathermy (electrocautery energy) to remove cancers in a more standardized en bloc resection where the tumor is removed as a whole, including the superior constrictor muscle, as part of a lateral oropharyngectomy (eFigure in Supplement 1).

The Postoperative Adjuvant Treatment for HPV-Positive Tumours (PATHOS) study^6^ is an international phase 3 trial designed to assess surgical margin safety limits in the context of deintensified adjuvant treatment. Participants recruited to PATHOS can receive either TLM or TORS (clinician choice), along with neck dissections if needed, before being randomized into the trial’s adjuvant therapy treatment groups.^7^

A consequence of the differences in surgical techniques means that TORS may offer greater margin clearance around tumors than TLM. The practical implications of greater margin clearance, effect on the prescription of adjuvant therapy, and ultimately oncological outcomes will be assessed when the PATHOS trial matures in 2027.

In this PATHOS substudy, which has been conducted independently of the defined trial objectives, we hypothesize and subsequently explore whether the different energy sources used and different surgical philosophy are associated with postoperative early swallowing function, feeding tube use, and specific factors related to quality of life. It is likely that additional head and neck–specific robotic platforms will be designed in the future. Any differences in functional outcomes following TLM or TORS may help in the design of a specific robot, given the unique anatomy and access constraints.

## Methods

### Study Design and Patients

**Figure 1.**
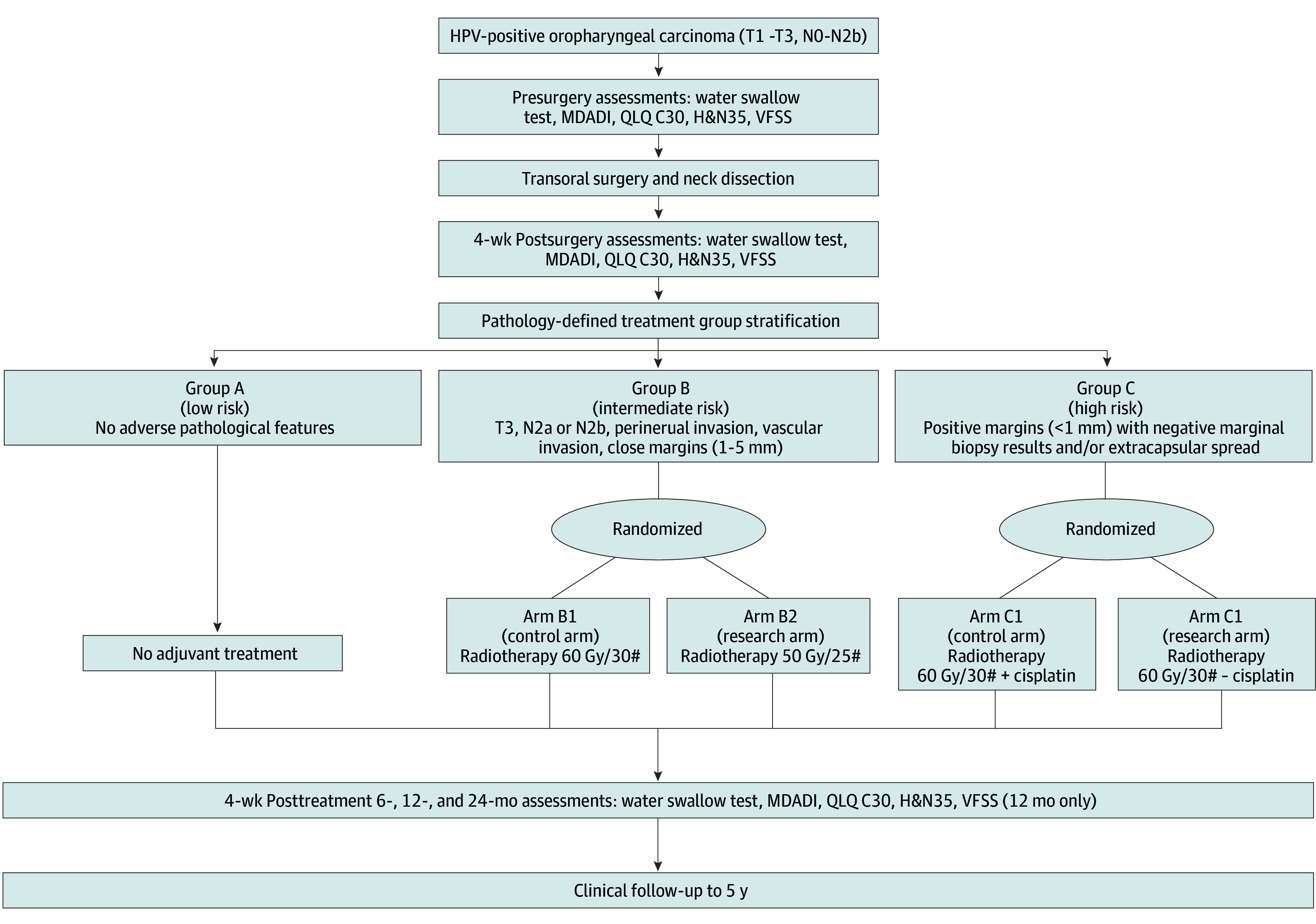
Postoperative Adjuvant Treatment for Human Papillomavirus (HPV)–Positive Tumours (PATHOS) Randomized Clinical Trial Schema HN&35 indicates 35-item European Organization for Research and Treatment of Cancer Head and Neck Questionnaire; MDADI, MD Anderson Dysphagia Inventory; QLQ CL30, 30-item European Organization for Research and Treatment of Cancer Quality of Life Questionnaire; and VFSS, videofluoroscopic swallow study. Number signs represent fractions of radiotherapy.

The PATHOS trial is an international, randomized, controlled, phase 3 clinical study of deintensified adjuvant treatment after transoral surgery in patients with HPV-positive OPSCC and has been described elsewhere (Figure 1).^7^ This study was approved by a UK ethics committee. Written informed consent was obtained from all participants prior to surgery. This study followed the Strengthening the Reporting of Observational Studies in Epidemiology (STROBE) guideline.

The analysis described herein is a planned substudy conducted prior to the end of full recruitment and focusing on prospectively collected data at the presurgical and 4-week postsurgical points, before the start of the randomized adjuvant treatment. The substudy protocol and statistical analysis plan were approved by the PATHOS Trial Management Group, including patient representatives.

Patients are eligible for the PATHOS trial if they have HPV-positive OPSCC, tested with p16 immunohistochemistry and confirmed by high-risk HPV in-situ hybridization, have undergone clinical staging with T1 to T3 N0 to N2b M0 (TNM7) results, and their primary tumor is considered resectable by transoral surgery by the local multidisciplinary team. This substudy was designed to use only data routinely collected in the PATHOS trial. No additional data were sought or collected. It aimed to compare the functional outcomes for the 4-week postoperative period following either TLM or TORS in the management of HPV-positive OPSCC by (1) describing the patient populations treated by TORS and TLM; (2) analyzing the length of in-hospital stay following surgery between patients treated with TORS and with TLM; (3) analyzing the use of and duration of nasogastric tube (NGT) feeding 4 weeks after surgery between patients treated with TORS and TLM; and (4) analyzing the patient-reported outcome measures, water swallow test results, and videofluoroscopy results at 4 weeks after surgery to assess pain and swallowing function between patients treated with TORS and with TLM.

In this substudy we only included patients in the PATHOS trial who underwent either TLM or TORS. Choice of TLM or TORS was at the discretion of the treating clinician. Neck dissection was undertaken as per standard protocols. Although permitted in the PATHOS protocol, we excluded patients who had a neck dissection after the primary surgery or who had a repeated resection of the primary tumor, to try to reduce factors other than surgical technique that may affect the outcomes in which we were interested. For our analyses of NGT use, we further excluded patients who had an NGT inserted prior to surgery.

Patients were clinically assessed with a timed 100-mL water swallow test and a videofluoroscopic swallow study (VFSS) (patients in the UK only), and completed patient-reported outcome measures (European Organization for Research and Treatment of Cancer [EORTC] 30-item Quality of Life Questionnaire [QLQ C30],^8^ EORTC 35-item Head and Neck questionnaire [H&N35],^9^ and the MD Anderson Dysphagia Inventory [MDADI]^10^) prior to and 4 weeks after surgery. Postoperative hemorrhage rates were also collected. Prior to performing the analysis, we defined the items within the QLQ C30 and H&N35 of clinical relevance to the study aims regarding function. These were the QLQ C30 global score, constipation subdomain (we hypothesized that this may reflect opiate analgesia use), and a summary score and the H&N35 swallowing, opening mouth, use of pain killers, and weight loss (participants’ weight measurements were not available before treatment) subdomains and a summary score. Minimum clinically important differences (MCID) of 5 to 10 points have been defined for the QLQ C30,^11^ although it is acknowledged that this may be too simplistic, does not differentiate between scales, and may not be achievable in all settings.^12^ For the H&N35, MCIDs range from 10 to 14 for the entire questionnaire,^13^ but the MCID for individual items has not been defined. The MDADI is a patient-reported swallowing outcome measure, specifically designed and psychometrically validated for the population with head and neck cancer, that reports global score, physical functioning, and composite score. The 19-item composite MDADI score at 1 year after treatment is included as a co–primary end point in PATHOS. The MCID for the MDADI questionnaire is often set at 10 points on the composite scale and was first described in a cross-sectional study of 1136 patients with head and neck cancer attending a modified barium swallow evaluation.^14^ The mean composite MDADI score was 64, and a 10-point difference differentiated patients who depended on feeding tubes from those who did not, and patients observed to aspirate from those who did not. Some observers have questioned whether 10 points represents the smallest difference that may be clinically important for high-functioning patients with HPV-positive OPSCC.^15^ The water swallow test measures swallow performance over time by measuring swallow capacity (milliliters per swallow) and volume (milliliters per second).^16^ An MCID of 4 mL/s on the water swallow test has been defined when applied to individual patients’ deterioration over 12 months.^17^ The VFSS was conducted by a speech and language therapist with the required level of competency as set out by the Royal College of Speech and Language Therapist guidelines and involved swallowing liquids and solids to a detailed assessment protocol specified in the PATHOS protocol. Video recordings of the VFSS were scored centrally by trained speech and language therapists (from the UK and US) who were blind to patient, treatment, and time point. This involved a Penetration-Aspiration Scale score (an 8-point, ordinal rating with ≥6 representing aspiration)^18^ and the Dynamic Imaging Grade of Swallowing Toxicity, version 2 criteria, a 5-point, ordinal rating of pharyngeal dysphagia with 2 or greater representing high-grade or moderate to severe dysphagia.^19,20^

### Statistical Analysis

All analyses were conducted using STATA, version 17 (StataCorp LLC), according to a statistical analysis plan written before any data were analyzed. The sample size was opportunistic based on recruitment into the PATHOS trial at the time the study was conceived, given the exploratory nature of the analysis. Length of hospital stay was calculated from date of surgery to date of discharge and compared between surgical techniques using univariable and multivariable Cox proportional hazards regression and including age, sex, smoking status, anatomical site, and T stage and treating center as a shared frailty. Rates of NGT insertion were compared between surgical techniques using univariable and multivariable logistic regression models (including age, sex, smoking status, anatomical site, and T stage) and a multilevel mixed model that also included treating center. For those who had an NGT inserted, length of tube insertion was compared between surgical techniques using the same methods as for length of hospital stay. Quality of life scores and water swallow test measures between baseline and 4 weeks after surgery were compared between surgical types using linear regression including baseline score only and multilevel mixed-effects linear regression including surgery type, baseline score, age, anatomical site, pathological T stage, sex, and smoking status and center as a second level. The 4-week postsurgical rates of aspiration and high-grade dysphagia from VFSS scores were compared between surgical types using multilevel mixed-effects logistic regression, including surgery type, age, anatomical site, pathological T stage, sex, and smoking status and center as a second level. Two-tailed *P* < .05 was taken as significant statistical evidence.

## Results

### Participants

**Figure 2.**
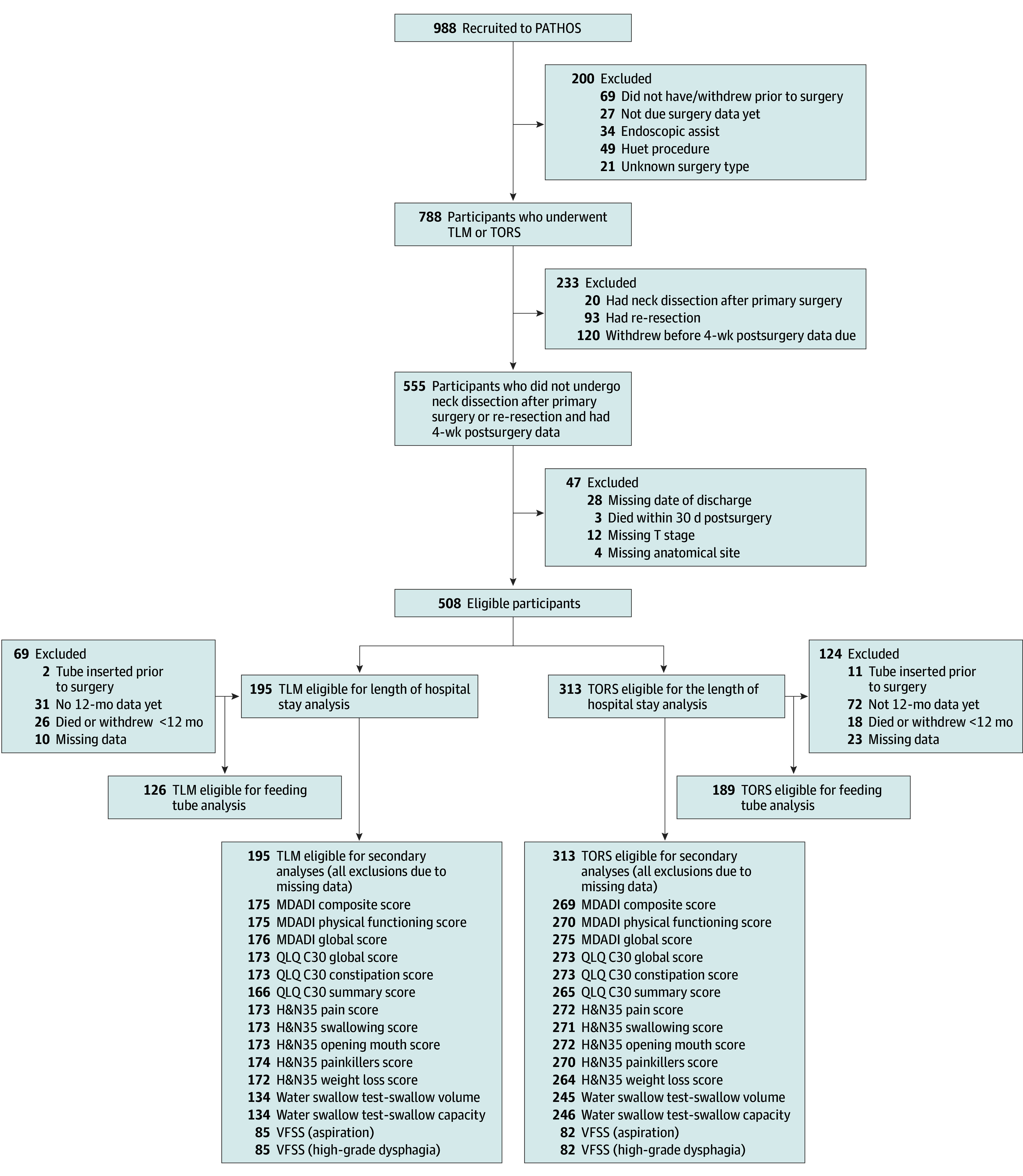
Flow Diagram for Substudy Analysis H&N35 indicates 35-item European Organization for Research and Treatment of Cancer Head and Neck Questionnaire; MDADI, MD Anderson Dysphagia Inventory; PATHOS, Postoperative Adjuvant Treatment for HPV (Human Papillomavirus)-Positive Tumours; QLQ CL30, 30-item European Organization for Research and Treatment of Cancer Quality of Life Questionnaire; TLM, transoral laser microsurgery; TORS, transoral robotic surgery; and VFSS, videofluoroscopic swallow study.

Figure 2 shows the flow of patients through the analyses. A total of 989 patients were recruited into the PATHOS trial from 40 centers in the UK, Germany, France, the US, and Australia between November 1, 2015, and August 31, 2023, when the data for this substudy were extracted. Of these, 794 patients had either TLM or TORS, and 508 (195 [38.4%] receiving TLM and 313 [61.6%] receiving TORS) were eligible and had complete data for the length of hospital stay analysis (118 [23.2%] female and 390 [76.8%] male; median age, 58.3 [IQR, 52.8-63.6] years). Fewer patients were available for the NGT analysis largely due to these data being collected at a later time (see Figure 2). Fewer patients were also available for the patient-reported outcome measures and water swallow analyses largely due to patients declining these nonroutine assessments.

Baseline, surgical procedure, and postoperative pathology data (Table 1) show very similar distributions for both TLM and TORS groups. There were more participants in the TORS group who received bilateral neck dissections than in the TLM group (54 of 313 [17.3%] vs 6 of 195 [3.1%]).

**Table 1.** Demographic and Disease Data

Characteristic	Treatment group, No. (%) of patients	
	TLM (n = 195)	TORS (n = 313)
Age at registration, median (IQR), y	57.7 (52.1-63.9)	58.8 (53.2-63.5)
Ratio of male to female	155:40	235:78
Smoking history		
Current	9 (4.6)	10 (3.2)
Former	92 (47.2)	127 (40.6)
Never	94 (48.2)	176 (56.2)
Surgical anatomical site		
Tonsil only	119 (61.0)	198 (63.3)
Base of tongue only	28 (14.4)	65 (20.8)
Posterior pharyngeal wall only	1 (0.5)	1 (0.3)
Tonsil and soft palate	11 (5.6)	6 (1.9)
Tonsil and vallecula	0	1 (0.3)
Tonsil and tongue	21 (10.8)	27 (8.6)
Tonsil and posterior pharyngeal wall	0	2 (0.6)
Vallecula and tongue	2 (1.0)	2 (0.6)
Tongue and posterior pharyngeal wall	1 (0.5)	1 (0.3)
Tonsil, soft palate, and tongue	6 (3.1)	3 (1.0)
Tonsil, soft palate, and posterior pharyngeal wall	1 (0.5)	0
Tonsil, vallecula, and tongue	2 (1.0)	1 (0.3)
Tonsil, vallecula, and posterior pharyngeal wall	0	1 (0.3)
Vallecula, tongue, and posterior pharyngeal wall	0	1 (0.3)
Tonsil, soft palate, tongue, and posterior pharyngeal wall	2 (1.0)	2 (0.6)
All above sites	1 (0.5)	2 (0.6)
Surgical anatomical site		
Lateral (tonsil and/or soft palate only)	130 (66.7)	204 (65.2)
Nonlateral	65 (33.3)	109 (34.8)
Surgery performed after diagnostic tonsillectomy		
Yes	45 (23.1)	81 (25.9)
No	150 (76.9)	232 (74.1)
Neck dissection		
Left only	86 (44.1)	123 (39.3)
Right only	101 (51.8)	136 (43.5)
Left and right	6 (3.1)	54 (17.3)
None	2 (1.0)	0
Pathological T stage		
T1	89 (45.6)	149 (47.6)
T2	92 (47.2)	153 (48.9)
T3	14 (7.2)	7 (2.2)
T4a	0	3 (1.0)
T4b	0	1 (0.3)
Pathological N stage		
N0	22 (11.3)	32 (10.2)
N1	45 (23.1)	87 (27.8)
N2a	48 (24.6)	80 (25.6)
N2b	76 (39.0)	112 (35.8)
N3	3 (1.5)	1 (0.3)
Missing	1 (0.5)	1 (0.3)

Abbreviations: TLM, transoral laser microsurgery; TORS, transoral robotic surgery.

### Length of Hospitalization

On univariable analysis, length of hospital stay appeared to be longer after TORS (median, 5 [95% CI, 5-6] days) than TLM (median, 3 [95% CI, 2-4] days) (median difference, 2.6 [95% CI, 1.8-3.5] days; hazard ratio [HR], 0.66 [95% CI, 0.55-0.79]) (eTable 1 in Supplement 1). This effect was maintained on multivariable analysis including age, sex, smoking status, anatomical site, and T stage but not when treating center was also included (HR, 0.89 [95% CI, 0.69-1.16]). In fact, no variables showed evidence of association with length of hospital stay once we accounted for treating center.

### Use of Feeding Tubes

On univariable analysis, NGT insertion rates were significantly higher after TORS (85 of 189 [45.0%]) than TLM (10 of 126 [7.9%]) (odds ratio [OR], 9.48 [95% CI, 4.68-19.22]) (Table 2). This association was maintained on multivariable analysis (same variables as above) and after treating center was also included (OR, 4.41 [95% CI, 1.01-19.32]). Those who were 65 years or older, female, and former smokers also showed higher odds of NGT insertion but only in the multivariable model that included center. For those who had an NGT, there was no difference in duration of NGT insertion (median, 5 [95% CI, 0.5-12] days for TLM vs 6 [95% CI, 4-6] days for TORS; multivariable HR, 1.05 [95% CI, 0.52-2.12]) (eTable 2 in Supplement 1).

**Table 2.** Factors Associated With Feeding Tube Inserted Within 4 Weeks After Surgery

Factor	No. (%) of patients		OR (95% CI)		
	Total included in analysis	Total with feeding tube fit	Univariable model	Multivariable model without center	Multilevel model with center as random effect
Surgery type					
TLM	126 (40.0)	10 (7.9)	1 [Reference]	1 [Reference]	1 [Reference]
TORS	189 (60.0)	85 (45.0)	9.48 (4.68-19.22)	9.90 (4.83-20.29)	4.41 (1.01-19.32)
Age, y					
<55	106 (33.7)	30 (28.3)	1 [Reference]	1 [Reference]	1 [Reference]
55-65	145 (46.0)	42 (29.0)	1.03 (0.59-1.80)	0.85 (0.46-1.58)	1.63 (0.61-4.36)
≥65	64 (20.3)	23 (35.9)	1.42 (0.73-2.76)	1.55 (0.73-3.28)	4.85 (1.25-18.85)
Sex					
Female	78 (24.7)	30 (38.5)	1.65 (0.97-2.83)	1.50 (0.82-2.74)	5.74 (1.83-18.04)
Male	237 (75.2)	65 (27.4)	1 [Reference]	1 [Reference]	1 [Reference]
Smoking status					
Never	173 (54.9)	54 (31.2)	1 [Reference]	1 [Reference]	1 [Reference]
Former	134 (42.5)	40 (29.9)	0.94 (0.57-1.53)	1.01 (0.58-1.76)	2.99 (1.11-8.09)
Current	8 (2.5)	1 (12.5)	0.31 (0.04-2.62)	0.29 (0.03-2.59)	0.43 (0.01-33.16)
Anatomical site					
Lateral	218 (69.2)	67 (30.7)	1 [Reference]	1 [Reference]	1 [Reference]
Nonlateral	97 (30.8)	28 (28.9)	0.91 (0.54-1.55)	0.81 (0.45-1.46)	1.21 (0.45-3.30)
T stage					
T1	140 (44.4)	44 (31.4)	1 [Reference]	1 [Reference]	1 [Reference]
T2+	175 (55.6)	51 (29.1)	0.90 (0.55-1.46	0.94 (0.55-1.61)	1.40 (0.57-3.44)

Abbreviations: NA, not applicable; OR, odds ratio; TLM, transoral laser microsurgery; TORS, transoral robotic surgery.

### Patient-Reported Outcomes and Water Swallow Test

Mean scores favored TLM (relative to TORS) with small effects in all investigated MDADI domains and the H&N35 swallowing item at 4 weeks after surgery (Table 3). Between-group difference was −4.89 (95% CI, −8.27 to −1.50) for MDADI composite score; −6.37 (95% CI, −10.15 to −2.59) for MDADI physical functioning score; −10.02 (95% CI, −16.50 to −3.54) for MDADI global score; and 7.24 (95% CI, 2.17-12.30) for H&N35 swallowing score. There were no clinically meaningful differences for QLQ C30 global, constipation, or summary scores and H&N35 pain, opening mouth, pain killers, or weight loss scores between the TORS and TLM groups. Water swallow test scores also favored TLM, but differences were not clinically significant.

**Table 3.** Quality of Life Scores, Water Swallow Test Measures, and Videofluoroscopy Rates of Aspiration and High-Grade Dysphagia at Baseline and 4 Weeks After Surgery by Surgery Type

Measure	Mean (SD) [median (IQR)]						Simple multivariable^a^		Full multivariable^b^	
	TLM group			TORS group						
	No. of patients	Baseline score	4-wk Postsurgical score	No. of patients	Baseline score, mean (SD) [median (IQR)]	4-wk Postsurgical score, mean (SD) [median (IQR)]	Effect of surgery, between-group difference (95% CI)^c^	*P* value	Effect of surgery, between-group difference (95% CI)^c^	*P* value
MDADI composite score^d^	175	91.3 (10.1) [95.8 (88.4 to 100)]	78.1 (14.4) [76.8 (68.4 to 91.6)]	269	89.7 (11.9) [95.8 (81.0 to 100)]	72.4 (15.9) [73.7 (61.1 to 83.2)]	−5.00 (−7.76 to −2.23)	<.001	−4.89 (−8.27 to −1.50)	.005
MDADI physical functioning score^d^	175	92.4 (12.5) [100 (87.5 to 100)]	74.6 (17.8) [75 (60 to 90)]	270	89.7 (14.9) [100 (80 to 100)]	67.7 (16.7) [67.5 (55.0 to 80.0)]	−6.04 (−9.18 to −2.89)	<.001	−6.37 (−10.15 to −2.59)	.001
MDADI global score^d^	176	91.1 (17.8) [100 (75 to 100)]	70.2 (29.0) [75 (50 to 100)]	275	88.2 (22.5) [100 (75 to 100)]	61.3 (31.3) [75 (25 to 75)]	−8.02 (−13.65 to −2.39)	.005	−10.02 (−16.50 to −3.54)	.002
QLQ C30 global score^e^	173	78.5 (18.3) [83.3 (66.7 to 91.7)]	64.7 (20.4) [66.7 (50.0 to 83.3)]	273	78.3 (18.5) [83.3 (66.7 to 91.7)]	64.8 (20.1) [66.7 (50.0 to 83.3)]	0.21 (−3.17 to 3.59)	.90	−0.30 (−4.65 to 4.05)	.89
QLQ C30 constipation score^f^	173	6.9 (18.1) [0 (0 to 0)]	24.1 (31.6) [0 (0 to 33.3)]	273	9.3 (21.1) [0 (0 to 0)]	24.5 (28.4) [33.3 (0 to 33.3)]	−0.09 (−5.66 to 5.49)	.98	2.19 (−4.46 to 8.85)	.52
QLQ C30 summary score^e^	166	88.6 (14.2) [94.0 (85.5 to 98.5)]	77.8 (17.6) [81.4 (70.6 to 90.6)]	265	88.2 (12.5) [92.1 (84.2 to 97.0)]	78.3 (15.5) [81.2 (70.8 to 90.2)]	0.74 (−1.83 to 3.31)	.57	0.39 (−2.61 to 3.40)	.80
H&N35 pain score^f^	173	14.6 (18.0) [8.3 (0 to 25.0)]	34.0 (25.6) [33.3 (16.7 to 50.0)]	272	17.5 (19.7) [8.3 (0 to 25.0)]	36.5 (23.0) [33. (19.4 to 50.0)]	1.48 (−2.91 to 5.87)	.51	4.58 (−0.90 to 9.96)	.10
H&N35 swallowing score^f^	173	5.6 (12.0) [0 (0 to 8.3)]	16.7 (18.6) [8.3 (0 to 25.0)]	271	6.8 (15.7) [0 (0 to 8.3)]	21.4 (22.9) [16.7 (8.3 to 33.3)]	4.25 (0.33 to 8.17)	.03	7.24 (2.17 to 12.30)	.005
H&N35 opening mouth score^f^	173	4.6 (16.6) [0 (0 to 0)]	30.8 (31.7) [33.3 (0 to 33.3)]	272	6.7 (17.4) [0 (0 to 0)]	33.2 (31.3) [33.3 (0 to 33.3)]	1.67 (−4.22 to 7.57)	.58	3.24 (−3.76 to 10.24)	.36
H&N35 use of pain killers score^f^	174	15.7 (16.7) [0 (0 to 33.3)]	23.6 (15.2) [33.3 (0 to 33.3)]	270	16.3 (16.7) [0 (0 to 33.3)]	23.2 (15.4) [33.3 (0 to 33.3)]	−0.44 (−3.31 to 2.42)	.76	−0.31 (−3.18 to 2.56)	.83
H&N35 weight loss score^f^	172	10.5 (15.5) [0 (0 to 33.3)]	15.1 (16.6) [0 (0 to 33.3)]	264	6.4 (13.2) [0 (0 to 0)]	13.5 (16.4) [0 (0 to 33.3)]	−1.15 (−4.33 to 2.02)	.48	0.11 (−3.83 to 4.06)	.96
Swallow volume, mean (SD) [median (IQR)], mL/swallow	134	22.7 (10.8) [20 (16.7 to 25.0)]	19.1 (9.1) [16.7 (12.5 to 25.0)]	245	22.2 (13.3) [20 (16.7 to 25.0)]	16.5 (10.9) [14.3 (11.1 to 20.0)]	−2.37 (−4.22 to −0.53)	.01	−1.56 (−4.01 to 0.88)	.21
Swallow capacity, mL/s	134	16.5 (8.3) [14.3 (11.1 to 20.0)]	13.0 (6.5) [12.5 (7.7 to 16.7)]	246	17.3 (10.0) [16.7 (11.1 to 20.0)]	12.0 (8.0) [11.1 (5.9 to 16.7)]	−1.32 (−2.72 to 0.07)	.06	−1.51 (−3.11 to 0.10)	.07
Aspiration rate, No. (%)	85	1 (1.2)	4 (4.7)	82	0	11 (13.4)	3.14 (0.96 to 10.29)	.06	3.29 (0.95 to 11.40)	.06
High grade dysphagia rate, No. (%)	85	0	7 (8.2)	82	1 (1.2)	15 (18.3)	2.49 (0.96 to 6.48)	.06	3.32 (0.81 to 13.56)	.10

Abbreviations: H&N35 indicates 35-item European Organization for Research and Treatment of Cancer Head and Neck Questionnaire; MDADI, MD Anderson Dysphagia Inventory; PATHOS, Postoperative Adjuvant Treatment for HPV (Human Papillomavirus)-Positive Tumours; QLQ CL30, 30-item European Organization for Research and Treatment of Cancer Quality of Life Questionnaire; TLM, transoral laser microsurgery; TORS, transoral robotic surgery.

^a^ Linear/logistic regression including surgery type and baseline score.

^b^ Multi-level mixed-effects linear/logistic regression including surgery type, baseline score, age, anatomical site, pathological t-stage, sex, smoking status, and center as a second level.

^c^ Linear regression coefficient except for rates where odds ratios are given.

^d^ Scores range from 20 (extremely low functioning) to 100 (high functioning).

^e^ Scores range from 0 to 100; a higher score represents a higher (better) level of functioning.

^f^ Scores range from 0 to 100; a higher score represents a higher (worse) level of symptoms.

### Videofluoroscopy

We analyzed 167 VFSSs (85 in the TLM group and 82 in the TORS group) conducted 4 weeks after surgery. There were large differences in rates of aspiration and high-grade dysphagia, with higher rates among the TORS group for aspiration (4 of 85 [4.7%] after TLM vs 11 of 82 [13.4%] after TORS; multivariable OR, 3.29 [95% CI, 0.95-11.40]) and high-grade dysphagia (7 of 85 [8.2%] after TLM vs 15 of 82 [18.3%] after TORS; multivariable OR, 3.32 [95% CI, 0.81-13.56]), but these differences were not significant; the 95% CIs were wide due to the smaller sample size for these outcome measures, and no definitive conclusion can be made. The amount of missing data was high for these end points because VFSS is only conducted in the UK and not internationally in PATHOS. In addition, there is a substantial time lag between the conduct of the assessment and central review to obtain scores, and reviews remain ongoing for the main trial outcomes. However, baseline variables were well balanced between those patients with and without missing data (eTable 3 in Supplement 1).

### Sensitivity Analysis

After finding an imbalance in the neck dissection variable at baseline, we conducted sensitivity analyses that additionally included the neck dissection variable in the multivariable models (eTable 4 in Supplement 1). The magnitude of the difference in outcomes between the 2 surgical types was broadly unaffected, apart from the effect on aspiration rate, which although still favoring TLM, became smaller (OR, 2.69 [95% CI, 0.72-10.10]), with the width of the 95% CI still preventing definitive conclusions about the true effect.

### Postoperative Hemorrhage Rates

Major (life threatening) postoperative hemorrhage occurred in 2 of 195 patients (1.0%) after TLM and 5 of 313 (1.6%) after TORS.

## Discussion

The PATHOS trial has presented a unique opportunity to compare 2 different transoral surgical techniques used in the management of HPV-positive OPSCC. In this cohort study, TORS was potentially associated with higher rates of NGT use and moderately worse H&N35 swallowing and MDADI scores at 4 weeks after surgery compared with TLM. Rates of aspiration and high-grade dysphagia favored TLM, but 95% CI were wide due to smaller sample sizes for those outcomes. The consistent trend across the range of patient-reported outcomes and VFSS and NGT use suggest a harmonized clinical picture that early postoperative functional outcomes were less impaired following TLM than TORS, although differences were relatively small and clinical relevance was uncertain. These findings could be of interest to colleagues considering the design of head and neck–specific robotic platforms.

Comparative studies of TLM and TORS for OPSCC are scarce. Sievert et al^21^ compared outcomes, primarily oncological outcomes, between 2003 and 2012 (30 patients with TLM and 24 with TORS). There were no clear differences observed between the 2 groups in terms of postoperative outcome measures. Parimbelli et al^22^ performed a cost utility analysis of TORS compared with TLM for OPSCC in 2 Swiss institutions. They concluded that TLM was more cost-effective than TORS to treat OPSCC. However, the analysis had an emphasis on surgical margins and how the techniques may then determine the need for radiotherapy and/or chemotherapy.

The results presented in this report suggest potential benefits of using laser energy and/or applying the transtumoral surgical philosophy adopted in a TLM approach as head and neck transoral–specific surgical robots are developed in the future. The lower rate of NGT use and reduced impairment of swallowing observed in participants treated with TLM may reflect the greater energy dispersion into soft tissues with TORS (electrocautery vs laser) and/or the routine resection of the superior constrictor muscle with TORS.

The observed difference in MDADI scores represents a small effect size with uncertain clinical meaning. It is possible that even a small difference in postsurgical function could result in better recovery prior to the start of adjuvant treatment and may translate into a larger effect after adjuvant therapy is delivered to the surgical bed. This remains speculative, and the long-term implications of the small functional difference seen acutely after treatment require further study.

### Strengths and Limitations

This is a large comparative study of functional outcomes following TORS vs TLM for HPV-positive OPSCC. The recruiting institutions’ practices clearly affected the length of hospital stay and NGT use and have been accounted for in the analysis. Data on individual clinicians’ practice were not available and may influence NGT use beyond that of the institutional practices, and the rationale for use was not documented or factored in. The data represent a nonrandomized analysis with multiple end point comparisons not adjusted for multiplicity. As such, the results should be seen as hypothesis generating rather than definitive. The unblinded nature of the study means that quality of life outcomes may be biased. Additionally, there is a lack of clarity as to the optimal MCID for the quality of life measures and water swallow tests that we used, especially given the particular patient context and fact that we were looking for between-group rather than within-patient differences. Furthermore, the study has focused solely on the early postoperative recovery period, at 4 weeks following surgery. It cannot comment on the effect of surgical philosophy (TORS vs TLM) on margins and how this may relate to the allocation of patients to the different adjuvant treatment after surgery. Furthermore, it cannot comment on the longer-term effects of TLM and TORS, in combination with adjuvant treatment, on function and quality of life outcomes. We plan to analyze longer-term effects when the PATHOS trial matures.

Due to the nature of the end points, some missing data are inevitable. This is particularly true for the VFSS scores, which were only obtained in the UK and for which there is a lag between the time of assessment and scoring as video recordings are collected and disseminated for review. However, our data show that the distribution of baseline characteristics was similar for those with and without scores, suggesting that bias may be minimal.

## Conclusions

The data in this cohort substudy of the PATHOS trial suggest that a TLM approach to HPV-positive OPSCC may cause moderately less impairment to swallowing and quality of life over the first 4 weeks following surgery when compared with a TORS approach. In most high-income countries of the world, many surgeons now choose to use TORS rather than TLM for the resection of HPV-positive OPSCC. This makes sense, as robots confer benefits with respect to tumor visualization, access, and training as well as ergonomic benefits to reduce tremor and enhance surgeon comfort. These findings may be of interest in the design and development of future head and neck–specific robotic platforms.

## Notes

Supplement 1. **eTable 1.** Median Length of Inpatient Hospital Stay After Surgery **eTable 2.** Median Duration of Feeding Tube Inserted Within 4 Weeks After Surgery **eTable 3.** Distribution of Baseline Data by Whether the Videofluoroscopy Data Was Missing or Not **eTable 4.** Results of Multivariable Models With and Without Neck Dissection Included as an Independent Variable **eFigure.** Tumour Removal Under TLM and TORS

Supplement 2.
Data Sharing Statement
