# Supplementary material for: Transoral Laser or Robotic Surgery Outcomes for Oropharyngeal Carcinoma: Secondary Analysis of the PATHOS Randomized Clinical Trial
Source: JAMA Otolaryngol Head Neck Surg. 2024 Oct 10;150(11):1002–11. doi: 10.1001/jamaoto.2024.3371 (PMC11581722; doi:10.1001/jamaoto.2024.3371)
Supplement: Supplement 1. — eTable 1. Median Length of Inpatient Hospital Stay After Surgery eTable 2. Median Duration of Feeding Tube Inserted Within 4 Weeks After Surgery eTable 3. Distribution of Baseline Data by Whether the Videofluoroscopy Data Was Missing or Not eTable 4. Results of Multivariable Models With and Without Neck Dissection Included as an Independent Variable eFigure. Tumour Removal Under TLM and TORS [file jamaotolaryngolheadnecksurg-e243371-s001.pdf]

Supplementary Online Content

O'Hara JT, Hurt CN, Ingarfield K, et al. Transoral laser or robotic surgery outcomes for oropharyngeal carcinoma: secondary analysis of the PATHOS randomized clinical trial. *JAMA Otolaryngol*. Published online October 10, 2024. This supplemental material has been provided by the authors to give readers additional information about their work.

doi: 10.1001/jamaoto.2024.3371

eTable 1. Median Length of Inpatient Hospital Stay After Surgery

eTable 2. Median Duration of Feeding Tube Inserted Within 4 Weeks After Surgery

eTable 3. Distribution of Baseline Data by Whether the Videofluoroscopy Data Was Missing or Not

eTable 4. Results of Multivariable Models With and Without Neck Dissection Included as an Independent Variable

eFigure. Tumour Removal Under TLM and TORS

This supplementary material has been provided by the authors to give readers additional information about their work.

© 2024 O'Hara JT et al. *JAMA Otolarygology.*

eTable 1. Median Length of Inpatient Hospital Stay After Surgery

		Length of hospital stay (days)		Univariable model		Multivariable model without centre		Multivariable model with centre as shared frailty	
		**n (%)**	**Median (95% CI)**	**HR (95% CI)**	**p-value**	**HR (95% CI)**	**p-value**	**HR (95% CI)**	**p-value**
**Surgery type**	TLM	195 (38.4%)	3 (2, 4)	1.00 (Ref.)	-	1.00 (Ref.)	-	1.00 (Ref.)	-
	TORS	313 (61.6%)	5 (5, 6)	0.66 (0.55, 0.79)	< 0.001	0.65 (0.54, 0.78)	<0.001	0.89 (0.69, 1.16)	0.401
**Age**	Less than 55 years	170 (33.5%)	4 (3, 5)	1.00 (Ref.)	-	1.00 (Ref.)	-	1.00 (Ref.)	-
	55 to 65 years	227 (44.7%)	4 (4, 5)	0.84 (0.69, 1.03)	0.087	0.84 (0.69, 1.02)	0.084	0.86 (0.70, 1.06)	0.155
	65 years and older	111 (21.9%)	6 (4, 7)	0.73 (0.57, 0.92)	0.009	0.71 (0.56, 0.91)	0.007	0.81 (0.63, 1.04)	0.104
**Sex**	Male	390 (76.8%)	4 (4, 5)	1.00 (Ref.)	-	1.00 (Ref.)	-	1.00 (Ref.)	-
	Female	118 (23.2%)	4 (3, 5)	1.26 (1.02, 1.55)	0.031	1.24 (1.00, 1.53)	0.050	1.16 (0.94, 1.45)	0.174
**Smoking status**	Never smoked	270 (53.2%)	4 (4, 5)	1.00 (Ref.)	-	1.00 (Ref.)	-	1.00 (Ref.)	-
	Ex-smoker	219 (43.1%)	4 (4, 5)	1.05 (0.88, 1.26)	0.571	0.99 (0.83, 1.19)	0.921	0.96 (0.79, 1.16)	0.657
	Current smoker	19 (3.7%)	3 (2, 5)	1.29 (0.81, 2.06)	0.280	1.21 (0.75, 1.93)	0.432	1.44 (0.89, 2.34)	0.137
**Anatomical site**	Lateral	334 (65.8%)	4 (4, 5)	1.00 (Ref.)	-	1.00 (Ref.)	-	1.00 (Ref.)	-
	Non-lateral	174 (34.3%)	4 (4, 5)	0.88 (0.73, 1.07)	0.196	0.90 (0.75, 1.09)	0.292	0.92 (0.75, 1.12)	0.390
**T stage**	T1	238 (46.9%)	4 (4, 5)	1.00 (Ref.)	-	1.00 (Ref.)	-	1.00 (Ref.)	-
	T2+	270 (53.2%)	5 (4, 5)	0.87 (0.73, 1.03)	0.109	0.86 (0.72, 1.03)	0.096	0.87 (0.73, 1.05)	0.153

© 2024 O'Hara JT et al. *JAMA Otolarygology.*

eTable 2. Median Duration of Feeding Tube Inserted Within 4 Weeks After Surgery

		Total participants who had a feeding tube	Length of tube feeding in days	Univariable model		Multivariable model without centre*	
		n	Median (95% CI)	HR (95% CI)	p-value	HR (95% CI)	p-value
**Surgery type**	TLM	10	5 (0.5, 12)	1.00 (Ref.)	-	1.00 (Ref.)	-
	TORS	85	6 (4, 6)	0.96 (0.50, 1.85)	0.894	1.05 (0.52, 2.12)	0.897
**Age**	Less than 55 years	30	5 (3, 7)	1.00 (Ref.)	-	1.00 (Ref.)	-
	55 to 65 years	42	4 (3, 6)	1.29 (0.80, 2.09)	0.291	1.54 (0.89, 2.67)	0.124
	65 years and older	23	7 (5, 14)	0.76 (0.44, 1.32)	0.332	0.87 (0.48 1.57)	0.640
**Sex**	Male	65	6 (4, 6)	1.00 (Ref.)	-	1.00 (Ref.)	-
	Female	30	5 (3, 8)	0.95 (0.61, 1.47)	0.809	0.90 (0.56, 1.45)	0.674
**Smoking status**	Never smoked	54	6 (5, 7)	1.00 (Ref.)	-	1.00 (Ref.)	-
	Ex-smoker	40	5 (2, 6)	1.25 (0.82, 1.90)	0.279	1.39 (0.86, 2.25)	0.362
	Current smoker	1	-	2.84 (0.38, 20.9)	0.307	2.58 (0.34, 19.8)	-
**Anatomical site**	Lateral	67	5 (4, 6)	1.00 (Ref.)	-	1.00 (Ref.)	-
	Non-lateral	28	6 (3, 7)	1.08 (0.69, 1.69)	0.732	1.11 (0.70, 1.75)	0.667
**T stage**	T1	44	5 (3, 6)	1.00 (Ref.)	-	1.00 (Ref.)	-
	T2+	51	6 (5, 7)	0.90 (0.60, 1.35)	0.612	0.93 (0.60, 1.44)	0.747

*Due to the small number of events it was not possible to include centre as a shared frailty. A multivariable cox model including surgery type and centre only was possible but did not alter the result for TORS: HR=0.92 (95% CI: 0.28-3.02, p=0.894).

© 2024 O'Hara JT et al. *JAMA Otolarygology.*

eTable 3. Distribution of Baseline Data by Whether the Videofluoroscopy Data Was Missing or Not

	**Not missing (n = 167)***	**Missing (n =341)***
**Age at registration** – n, median (IQR)	167, 58.2 (52.8, 63.6)	341, 58.3 (52.9, 64.2)
**Sex**		
Male	127 (76.0)	263 (77.1)
Female	40 (24.0)	78 (22.9)
**Smoking history**		
Current smoker	3 (1.8)	16 (4.7)
Ex smoker	72 (43.1)	147 (43.1)
Never smoked	92 (55.1)	178 (52.2)
**Surgical anatomical site - grouped**		
Lateral (tonsil/soft palate only)	112 (67.1)	222 (65.1)
Non-lateral	55 (32.9)	119 (34.9)
**Surgery performed after diagnostic tonsillectomy?**		
Yes	34 (20.4)	92 (27.0)
No	133 (79.6)	249 (73.0)
**Was a neck dissection done?**		
Left only	66 (39.5)	143 (41.9)
Right only	75 (44.9)	162 (47.5)
Left and right	26 (15.6)	34 (10.0)
No	0 (0.0)	2 (0.6)
**Pathological T stage**		
T1	75 (44.9)	163 (47.8)
T2	84 (50.3)	161 (47.2)
T3	8 (4.8)	13 (3.8)
T4a	0 (0.0)	3 (0.9)
T4b	0 (0.0)	1 (0.3)
**Pathological N stage**		
N0	20 (12.0)	34 (10.0)
N1	33 (19.8)	99 (29.0)
N2a	46 (27.5)	82 (24.0)
N2b	66 (39.5)	122 (35.8)
N3	2 (1.2)	2 (0.6)
Missing	0 (0.0)	2 (0.6)

*n (%) except where indicated

© 2024 O'Hara JT et al. *JAMA Otolarygology.*

eTable 4. Results of Multivariable Models With and Without Neck Dissection Included as an Independent Variable

	Full multivariable without neck dissection[A]		Full multivariable with neck dissection	
	Effect of surgery[B] (95%CI)	p-value	Effect of surgery[B] (95%CI)	p-value
**Median length of inpatient hospital stay post-surgery**	HR=0.89 (0.69, 1.16)	0.401	HR=0.89 (0.68, 1.16)	0.396
**Use of tube feeding inserted within 4 weeks post-surgery**	OR=4.41 (1.01, 19.3)	0.049	OR=4.50 (1.03,19.7)	0.045
**Median duration of feeding tube inserted within 4 weeks post-surgery**	1.05 (0.52, 2.12)	0.897	HR=1.02 (0.51, 2.07)	0.949
**MDADI Composite (score)**	-4.89 (-8.27, -1.50)	0.005	-5.16 (-8.57, -1.76)	0.003
**MDADI Physical Functioning (score)**	-6.37 (-10.15, -2.59)	0.001	-6.53 (-10.36,-2.70)	0.001
**MDADI Global (score)**	-10.02 (-16.50, -3.54)	0.002	-9.82 (-16.41,-3.23)	0.003
**EORTC C30 Global (score)**	-0.30 (-4.65, 4.05)	0.893	-0.80 (-4.47, 4.31)	0.972
**EORTC C30 Constipation (score)**	2.19 (-4.46, 8.85)	0.518	1.42 (-5.17, 8.00)	0.673
**EORTC C30 Summary (score)**	0.39 (-2.61, 3.40)	0.798	0.40 (-2.74, 3.54)	0.802
**EORTC H&N35 Pain (score)**	4.58 (-0.90, 9.96)	0.095	4.55 (-0.86, 9.96)	0.099
**EORTC H&N35 Swallowing (score)**	7.24 (2.17, 12.30)	0.005	7.11 (2.02, 12.20)	0.006
**EORTC H&N35 Opening Mouth (score)**	3.24 (-3.76, 10.24)	0.364	3.37 (-3.70, 10.45)	0.350
**EORTC H&N35 Pain Killers (score)**	-0.31 (-3.18, 2.56)	0.833	-0.06 (-3.07, 2.95)	0.970
**EORTC H&N35 Weight Loss (score)**	0.11 (-3.83, 4.06)	0.956	0.09 (-3.86, 4.04)	0.965
**Swallow Volume (mL/swallow)**	-1.56 (-4.01, 0.88)	0.210	-1.50 (-3.97, 0.96)	0.231
**Swallow Capacity (mL/second)**	-1.51 (-3.11, 0.10)	0.067	-1.41 (-3.05, 0.22)	0.090
**Aspiration rate – n(%)**	OR=3.29 (0.95, 11.4)	0.061	OR=2.69 (0.72, 10.1)	0.141
**High grade dysphagia rate – n(%)**	OR=3.1 (0.81, 13.61)	0.096	OR=4.19 (0.78, 22.5)	0.094

[A] Same results as presented in earlier tables to enable comparison

[B] linear regression coefficient except where indicated

© 2024 O'Hara JT et al. *JAMA Otolarygology.*

eFigure. Tumour Removal Under TLM and TORS

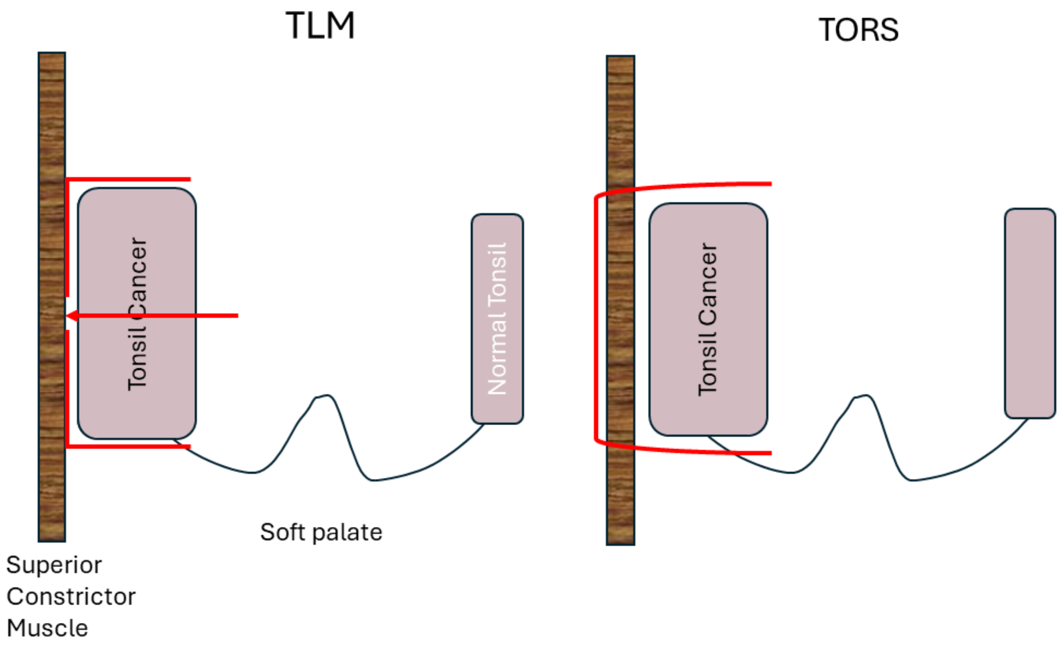

© 2024 O'Hara JT et al. *JAMA Otolarygology.*
