# Supplementary material for: Transoral Laser or Robotic Surgery Outcomes for Oropharyngeal Carcinoma: Secondary Analysis of the PATHOS Randomized Clinical Trial
Source: JAMA Otolaryngol Head Neck Surg. 2024 Oct 10;150(11):1002–11. doi: 10.1001/jamaoto.2024.3371 (PMC11581722; doi:10.1001/jamaoto.2024.3371)
Supplement: Supplement 2. — Data Sharing Statement [file jamaotolaryngolheadnecksurg-e243371-s002.pdf]

Data Sharing Statement

O'Hara. Transoral Laser or Robotic Surgery Outcomes for Oropharyngeal Carcinoma. *JAMA Otolaryngol Head Neck Surg*. Published October 10, 2024. doi:10.1001/jamaoto.2024.3371

Data
Data available: Yes
Data types: Deidentified participant data, Data dictionary
How to access data: CTRDataSampleRequests@cardiff.ac.uk
When available: With publication

Supporting Documents
Document types: None

Additional Information
Who can access the data: The PATHOS trial is not due to complete until 2028 and data release possibility before then will be limited and require IDMC and Sponsor approval. However, the Centre for Trials Research is a signatory of AllTrials and aims to make its research data available wherever possible, subject to regulatory approvals, any terms and conditions placed upon us from external providers, patient confidentiality and all laws concerning the protection of personal information. De-identified participant data and study documents (such as the study protocol, statistical analysis plan, participant information sheet, and informed consent form) are generally freely available, but recipients are expected to acknowledge the original creators in any public use of the data or in publishing research results based wholly or in part upon the data – anyone requesting access to data will be asked to agree to the terms of the Creative Commons Attribution 4.0 license. We may ask the requestor to cover reasonable cost for preparing and providing the data (for example physical storage and postage, where dataset size makes it impractical to provide data by electronic means). Please send requests for access to our data to the open data team (CTRDataSampleRequests@cardiff.ac.uk) for assessment, providing: • sufficient detail to uniquely identify the dataset sought. • appropriate contact details for the requestor.
Types of analyses: See above
Mechanisms of data availability: See above
Any additional restrictions: See above
